# Synthesis and Characterization of Novel D-A Type Neutral Blue Electrochromic Polymers Containing Pyrrole[3-c]Pyrrole-1,4-Diketone as the Acceptor Units and the Aromatics Donor Units with Different Planar Structures

**DOI:** 10.3390/polym11122023

**Published:** 2019-12-06

**Authors:** Haoguo Yue, Lingqian Kong, Bo Wang, Qing Yuan, Yan Zhang, Hongmei Du, Yunyun Dong, Jinsheng Zhao

**Affiliations:** 1 Shandong Key Laboratory of Chemical Energy Storage and Novel Cell Technology, Liaocheng University, Liaocheng 252059, China; haoguoyue@163.com (H.Y.); wangbo@lcu.edu.cn (B.W.); yuanqing@lcu.edu.cn (Q.Y.); zy@lcu.edu.cn (Y.Z.); duhongmei@lcu.edu.cn (H.D.); dongyunyun@lcu.edu.cn (Y.D.); 2 College of Chemistry and Chemical Engineering, Liaocheng University, Liaocheng 252059, China; 3 Department of chemistry, Dongchang College, Liaocheng University, Liaocheng 252059, China; lingqiankong@126.com

**Keywords:** electrochromic, pyrrole[3-c]pyrrole-1,4-diketone, donor-acceptor, neutral blue color, conjugated polymer

## Abstract

Three soluble conjugated polymers, named BEDPP, FLDPP, and CADPP, were prepared through the Suzuki polymerized reaction, and employed benzene (BE), fluorene (FL), and carbazole (CA) as the donor units, respectively. The electron-deficient molecule 2,5-bis-(2-octyldodecyl)-3,6-bis-(5-bromo-thiophene)-pyrrole[3-c]pyrrole-1,4-diketone(DPP) was introduced and used as the acceptor unit. The properties of these three copolymers were studied by a series of detailed characterization analysis, including X-ray photoelectron spectroscopy (XPS), colorimetry, electrochemical measurements, spectroelectrochemistry, kinetics, quantitative calculation, and thermogravimetric (TG) analysis, etc. The results revealed that BEDPP displayed a blue color in the neutral state and a light brown color in the oxidized state, FLDPP exhibited a cyan color in the neutral state and a gray color in the oxidized state, while CADPP displayed pure blue color in the neutral state and a light gray color in the oxidized state. All these polymers possess narrow optical band gaps lower than 1.80 eV and satisfactory thermal stability. The kinetic characterization showed that the optical contrasts (Δ*T*%) in the near-infrared region were superior to the visible region. The optical contrasts of BEDPP, FLDPP, and CADPP are 41.32%, 42.39%, and 45.95% in the near-infrared region, respectively, which made them a good application prospect in the near-infrared region. Amid the three polymers, CADPP has the highest coloration efficiency (around about 288 cm^2^·C^−1^) and fast switching times (0.77 s in the coloring process and 0.52 s in the bleaching process) in the visible region, and the comprehensive performance of CADPP can be comparable to that of the reported D-A (Donor-Acceptor) type blue color polymers. In general, based on the good performances and the stable neutral blue color, the three polymers had profound theoretical significance for the development of electrochromic material and the completion of the RGB (Red, Green, Blue) color space.

## 1. Introduction

Conjugated polymers have been considered as promising materials holding excellent optical and electrical properties with the charming benefits of being solution-processable, lightweight, having a facile synthesis, and good flexibility [1]. These characteristics make them a rapid development in the fields of organic field-effect transistors (OFET) [2], electrochromic devices [3], organic Li-ion batteries [4], solar cells [5], polymer light-emitting diodes (PLEDs) [6], and so on. Being an important research field of conjugated polymers, the electrochromism (EC) material can generate stable and reversible changes in color and transmittance with the application of an external voltage, which has attracted extensive explorations [7]. Compared with the traditional inorganic electrochromic materials, the advantages of conjugated polymers include higher coloration efficiency [8], shorter switching times, multiple colorations, fine-tunability of the band gap, feasibility of large-scale device production [9], and thin film flexibility [10]. Based on these advantages of practical value, electrochromic copolymers have achieved widespread applications encompassing smart windows [11], e-billboards [12], camouflage electrochromic fabrics [13], photodetectors [14], and so on.

It is well known that the exploration of novel high-performance electrochromic materials and multicolor changes are still the significant targets of scientists in the fields of electrochromic applications. Up to now, there have been several strategies to adjust the polymer colors, such as the increase of conjugated chain or alkyl chain [15], steric-hindrance effect [16], and donor-acceptor (D-A) [17] approach, among them, and the D-A approach is the most satisfactory and effective method for obtaining low band gap polymers. The polymers of the donor-acceptor (D-A) structure can modify the band gap effectively, relying on the structure resonance and intramolecular charge transfer [18]. Especially, the well-matched donor units and acceptor units are rather popular for low band-gap polymers. Among of them, the electron-rich units can elevate the highest occupied molecular orbital (HOMO) energy level and make the oxidation process becoming easier. Meanwhile, the electron-deficient unit can decrease the lowest unoccupied molecular orbital (LUMO) energy level and promote the reduction process as being more likely to happen [19].

The preparation of the blue component possesses the indispensable effect in the additive primary colors RGB (red, green, blue) with non-emission characteristics, which is the focus of electrochromic device applications [20,21]. Interestingly, all colors of polymers can be obtained by the mixture of three additive colors according to the theory of color mixing, which is very important for the preparation of simple colored electrochromic displays [22]. Compared with the neutral green color polymers, which requires two absorption bands (red and blue light) in the visible region, the polymers of neutral blue just need a single absorption band, which requires a maximum absorption peak in the red and orange region [9,22]. However, the neutral blue conjugated polymers with high-performance are not aplenty [23]. It is noteworthy to mention that the Cihaner group has reported a polymer named PProDOS-Np2 (pure blue to transparent) which has the high optical contrasts (84%) and outstanding stability [24]. Xu’s group reported a series of polymers (blue to transmissive) based on benzotriazole, and examined the effects of the side chains. The results showed that long alkyl chain can accelerate the switching speed in the visible and near-infrared regions [23]. Meng’s research team synthesized soluble neutral blue polymers based on thieno[3,2-b]thiophene. The addition of benzene and thiophene rings as the substitutions on the 3,6-position of the thieno[3,2-b]thiophene core improved the optical contrasts and stability, and concluded that different substitution units could be a subtle modification method for electrochromic behaviors [9]. Our group synthesized some satisfactory electrochromic materials, such as a neutral green polymer containing indolo[3,2-b]carbazole donor and diketopyrrolopyrrole acceptor [15], and red color material based on benzotriazole, quinoxaline, and benzene units [25]. Despite great efforts having been devoted to the study of multicolor copolymers, the numbers of high-performance blue color materials that are suitable for additive primary colors (RGB) are rare. Therefore, we were motivated to explore the blue polymers with excellent comprehensive properties and promising applications.

As one of several excellent commercial pigments, the high mobility, broad absorption range and great optoelectronic properties of pyrrole[3-c]pyrrole-1,4-diketone (DPP) containing polymers make it a well-received material in the fields of organic field-effect transistors, photovoltaic devices, and electrochromic devices [26,27]. The –NH bond in the DPP structure generates the intermolecular hydrogen bonds with adjacent carbonyl oxygen atoms, and the conjugated structure of lactam caused the strong electron affinity. Furthermore, the molecular orbital overlaps between different molecular layers produced π-π* interactions, and improved the mobilization of holes and electrons [27,28]. The results from studies show that the DPP acceptor unit can be aggregated with numerous different donor units on account of the ability of strong electron-withdrawing, which have been reported by Xu’s group, and the bluish-black hue polymers in neutral state could be available based on DPP and Propylenedioxythiophene (ProDOT) units [29]. Tieke’s research team found that only the 3,6-positon on the structure of cross-linked DPP polymers could link the thiophene unit and generate the desired conjugation sequences [30]. Lim’s group demonstrated the novel outstanding material named LGC-D148 which contains a diketopyrrolopyrrole (DPP) as the acceptor and 2,5-difluorophenylene-dithiophene as the donor, the films having a coloration efficiency of >900 cm^2^/C accompanying the color changes from green to transparent. These achievements indicated that diketopyrrolopyrrole unit is a promising acceptor for the construction of valuable electrochromic materials with the different donor units [31]. For the donor units, exploiting the inherent planarity and conjugated structure, a benzene ring could bridge the polymer and facilitate the charge transformation [32]. As the redox-active chromophores, the alkyl-substituted fluorene and carbazole were always used as the donor units to build D-A type electrochromic polymers [33]. Therein, alkylation of fluorene possesses high solubility in the organic solvent and stabilize on the carbon atom at the 9-position [34]. Although the electrochromism performances of carbazole-containing polymers have not been extensively studied, carbazole donors with the advantages of their high stability and ease of functionalization making them attractive EC donor materials [35]. Moreover, carbazole derivatives have multifunctional positions, including the 2,7-position, 3,6-position, and N-position. The introduction of long alkyl side chains on the N-position could produce good solubility, and the 2,7-position linked carbazole could achieve a lower band gap [36].

In accordance with the above studies, in this study we selected 2,5-bis(2-octyldodecyl)-3,6-bis (5-bromothiophene-pyrrole-pyrrole)-1,4-diketone as the acceptor unit, and benzene, fluorine, and carbazole as the donor units, respectively. Three polymers were synthesized successfully via the Suzuki coupling reaction, and the corresponding polymers named BEDPP, FLDPP, and CADPP. Through a series of detailed measurements, we found that the polymer films have a reversible electrochromism switching under a low driving potentials (<1.10 V), and the performances of fast switching speed, satisfactory color efficiency, and good thermal stability indicated that these copolymers were expected to be useful for practical value in the fields of electrochromic display applications. The properties of the three polymers have been investigated in detail and presented herein.

## 2. Experimental

### 2.1. Materials

Tetrakis (triphenylphosphine) palladium(Pd(pph_3_)_4_(99%) was purchased from commercial resources (Alfa Aesar Chemical ReagentCo.Ltd, Shanghai, China). 1,4-di(boronic acid pinacol)- benzene (>98%), 2-octyl dodecyl bromide (97%), 9,9-dioctylcarbazole-2,7-di(boronic acid pinacol)(>98%), 9,9-dioctyl fluorene-2,7-di(boronic acid pinacol) (>98%) were purchased from commercial resources (Soochiral Chemical Science Technology Co.Ltd, Suzhou, China). 3,6-dithiophen-2,5-dihydropyrrole[3-c]pyrrole-1,4-diketone (99%) were purchased from commercial resources (Derthon Optoelectronic Material Science Technology Co.Ltd, Shenzhen, China). High-performance chromatography of pure acetonitrile (ACN, 99.9%), anhydrous potassium carbonate (99.0%, K_2_CO_3_) and anhydrous magnesium sulfate (98%, MgSO_4_) were purchased from commercial resources (Tianjin Kermel Europe Co. Ltd, Tianjin, China). Tetrabutylammonium hexafluorophosphate (TBAPF_6_, 98%), N-bromosuccinimide (NBS, 98%), and N-N-dimethylamine (98%) were purchased from commercial resources (Aladdin Co. Ltd, Shanghai, China). Methanol (99.5%), methylene chloride (99.0%), chloroform (99.0%), acetone (99.5%), and alcohol (95%) were purchased from the commercial resources (Laiyang Fine Technology Development Zone Fine Chemical Co.Ltd, Yantai, China). The above reagents are used directly without other treatment. Indium-tin-oxide (ITO) coated glasses (sheet resistance: <10 ohm/sq) were purchased from commercial resources (Zhuhai Kaivo Optoelectronic Technology Co.Ltd, Zhuhai, China).

### 2.2. Instrumentation

Synthesis: Reaction bottle, gas device, chromatographic column, rotary evaporation device, cooling circulation pump, and a soxhlet extractor were used for the synthesis and purification of monomers and polymers.

Characterization: ^1^H NMR and ^13^C NMR spectroscopy were carried out on an Advance NEO 500 MHz spectrometer (Bruker BioSpin International AG, Zug, Switzerland), and used tetramethylsilane as the internal standard. Fourier transform infrared spectroscopy (FT-IR) (Thermo Nicolet Co., Madison, WI, USA) was recorded in a Nicolet 6700 FT–IR spectrometer, and used potassium bromide as a carrier. Pyris Diamond (TGA, TG, NETZSCH Scientific Instruments Trading Ltd., Germany) was employed to monitor the thermogravimetric with differential scanning calorimeter polymer under the nitrogen protection. The heating rate was 10 °C/min until the temperature up to 800°C in the whole procedure. X-ray photoelectron spectroscopy (XPS) analysis of the polymer films were measured on a Thermo Escalab Xi^+^ with a monochromated Al X-ray resource (Thermo Fisher Scientific, Waltham, MA, USA). A Shanghai Chen Hua CHI 760D electrochemical workstation (Chen Hua Instrument Co., Ltd, Shanghai, China) was chosen to detect the electrochemical properties of the polymers that were sprayed on the ITO (Indium Tin Oxides) glass. In a three-electrode system, conductive glass was used as the working electrode, and silver wire (0.02 V vs saturated calomel electrode (SCE)) was selected as the pseudo-reference electrode while platinum wire was chosen as the counter electrode. The spectral electrochemical and colorimetry tests of the polymer films were investigated by the Varian Cary 5000UV–VIS–IR spectrophotometer (Agilent Technologies Ltd, Mulgrave, Australia) and CHI 760 electrochemical analyzer (Chen Hua Instrument Co., Ltd, Shanghai, China). Digital photographs were taken by a Canon Power Shot A3000 IS digital camera. The copolymer films were obtained through the spay-casting method. Among them, the polymer was dissolved in chloroform, and stirred for 24 h by employing the electromagnetic stirrer to ensure the polymer dissolved completely. The prepared solution was sprayed on ITO conductive glass with a spray gun which characterized the polymers. GPC analyses were carried out on a Waters 2414 HPLC unit (Waters Inc., Milford, MA, USA) with a polystyrene gel column (Agilent PL gel 5μm, MIXEDC) at room temperature. A polystyrene standard kit was used for calibration, and chloroform was used as the eluent.

### 2.3. Synthesis Procedures

#### 2.3.1. Synthesis of Compound 2,5-Bis(2-Octyldodecyl)-3,6-Dithiophene-Pyrrole[3-c]Pyrrole-1,4-Diketone (B)

**Scheme 1:**
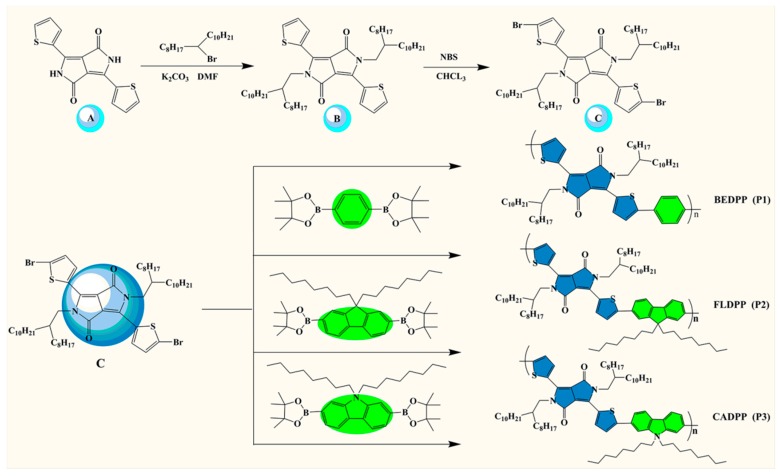
Synthetic route of monomer C (acceptor) and polymers BEDPP, FLDPP and CADPP.

2,5-bis(2-octyldodecyl)-3,6-dithiophene-pyrrole[3-c]pyrrole-1,4-diketone(B)wassynthesized according to the previous literature [37], and the synthetic routes were showed in Scheme 1. The specific operation as follows: the dried round-bottom flask (500 mL) was assembled with a condenser and protected by argon environment. 3,6-dithiophen-2,5-dihydropyrrole(3-c)-pyrrole-1,4-diketone (compound A, 6 g, 0.02 mol), 2-octyldodecyl-bromide (21.6 g, 0.06 mol), 160 mL N-N dimethylformamide and anhydrous potassium carbonate (11.16 g, 0.08 mol) were added to the reaction bottle in turn, then the mixture was stirred and refluxed under the temperature of 145 °C. The system was cooled to room temperature after reacted 15 h, the appropriate amount of deionized water and dichloromethane were added to the reaction bottle, which was used as the extractant to extract the compound B, and then we employed appropriate anhydrous magnesium sulfate to dry the deionized water, and removed all organic reagents by rotary evaporation under reduced pressure. The crude product was purified by silica gel column chromatography and got the dark red solid product (B) in the end. Yield: 76%. The ^1^H and ^13^C spectra of the compound B are shown in supplementary material Figure S1. The symbol δ represents chemical shift and related information of absorption peaks.

^1^H NMR (CDCl_3_, 500 MHz, ppm): δ = 8.88(d, 2H), 7.62(d, 2H), 7.26(t, 2H), 4.01(d, 4H), 1.90(s, 2H), 1.20–1.29(m, 64H), 0.88(m, 12H).

^13^C NMR (CDCl_3_, 101 MHz, ppm): δ = 161.72, 140.39, 135.23, 130.46, 129.81, 128.38, 107.88, 46.18, 37.71, 31.90, 31.12, 30.01, 29.50, 26.18, 22.69, 14.13.

#### 2.3.2. Synthesis of monomer2,5-Bis(2-Octyldodecyl)-3,6-Bis(5-Bromothiophene)-Pyrrole[3-c]Pyrrole-1,4-Diketone (C)

Compound B (3.27 g, 0.038 mol) and N-butylbromidediimide (1.9 g, 0.1076 mol) were added in a round-bottom flask (500 mL) that containing trichloromethane (100 mL), then light stirring reacted for 24 h under the condition of environment temperature 0 °C. After the reaction, the reacted solvent was removed by rotary evaporation under the reduced pressure and obtained the crude product. We used the silica gel to mix the crude material, and employed the n-hexane/dichloromethane (4:1 *v*/*v*) as the eluent to purify the product. Finally, the deep red solid (C) was obtained after purification by the chromatographic column. Yield: 79%. The ^1^H and ^13^C spectra of monomer C are shown in supplementary material Figure S2.

^1^H NMR (500MHz, CDCl_3_, ppm): δ = 8.64(d, 2H), 7.22 (d, 2H), 3.92 (d, 4H), 1.87 (s, 2H), 1.20–1.29 (m, 64H), 0.88(m, 12H).

^13^C NMR (101 MH_Z_, CDCl_3_, ppm): δ = 161.36, 139.37, 135.34, 131.41, 131.13, 118.96, 107.95, 46.30, 37.73, 31.88, 31.11, 29.56, 29.30, 26.14, 22.70, 14.14.

#### 2.3.3. Synthesis and Purification of Polymers

BEDPP: The specific synthesis route of the BEDPP polymer is shown in Scheme 1. The monomer C (500 mg, 0.49 mmol), 90 mL of toluene, 2M sodium carbonate solution (60 mL), tetrakis (triphenylphosphine) palladium(Pd(pph_3_)_4_, 25 mg, 0.02 mmol), tetrabutyl ammonium fluoride (13 mg, 0.05 mmol) and 1,4-di(boronic acid pinacol)-benzene (162 mg, 0.49 mmol) were added into a round-bottom flask. The whole reaction procedure takes 72 h under the protection of argon atmosphere. At the same time, the reaction temperature should stay at 90 °C. Next, we used the distilled water to extract carbonate (4–6 times) after the end of the reaction, and employ the anhydrous magnesium sulfate to remove all of the water mixed into the organic layer. Then the magnesium sulfate solid was removed by the method of extraction, and the crude product can be obtained by rotary distillation under the reduced pressure. The crude material was placed in a soxhlet extractor and purified with n-hexane, methanol, and acetone, in turn, and the extraction time of every solvent was24 h. The purified products were stored for characterization after vacuum drying at 60 °C. Yield: 72%. The ^1^HNMR spectra of BEDPP was shown in supplementary material Figure S3a. BEDPP: ^1^H NMR (500 MHz, CDCl_3_, ppm): δ = 8.56 (s, 2H), 8.01(s, 2H), 7.48 (s, 2H), 7.26 (s, 38H), 7.06 (d, 2H), 2.98 (s, 2H), 2.08 (d, 2H), 1.54 (m, 48H), 1.36 (s, 42H), 1.18 (m, 3H), 0.88 (t, 12H). The degree of polymerization was estimated from three parameters including the weight-average molecular weight (*M*_w_), the number-average molecular weight (*M*_n_), and the polymer dispersity index (PDI). For BEDPP, *M*_n_ = 21.8 k, *M*_w_ = 29.4 k, PDI = 1.35.

FLDPP was synthesized by the same procedure as for BEDPP. Compounds used: monomer C (500 mg, 0.49 mmol), 9,9-dioctylfluorene-2,7-di(boronicacid pinacol) (315 mg, 0.49 mmol). The amount of other reagents was remained the same as BEDPP. The ^1^HNMR of FLDPP was shown in supplementary material Figure S3b. FLDPP: ^1^H NMR (500 MHz, CDCl_3_, ppm): δ = 8.59 (s, 2H), 8.10 (s, 2H), 7.57 (s, 1H), 7.46 (m, 2H), 7.05 (s, 2H), 2.89 (s, 3H), 2.16 (t, 2H), 2.03 (s, 2H), 1.54 (d, 52H), 1.37 (m, 46H), 1.13 (m, 4H), 0.87 (m, 6H). The information on the molecular weight of FLDPP: *M*_n_ = 23.5 k, *M*_w_ = 33.4 k, PDI = 1.42.

CADPP was synthesized by the same procedure as for BEDPP. Compounds used: monomer C (500 mg, 0.49 mmol), 9,9-dioctylcarbazole-2,7-di(boronic acid pinacol) (323 mg, 0.49 mmol). The amount of other reagents was remained the same as BEDPP. The ^1^HNMR of CADPP was shown in supplementary material Figure S3c. CADPP: ^1^H NMR (500 MHz, CDCl_3_, ppm): δ = 8.69 (s, 2H), 8.26 (s, 2H), 7.85 (s, 3H), 7.47 (s, 2H), 7.06 (s, 2H), 4.05 (s, 4H), 3.47 (s, 4H), 2.16 (m, 3H), 2.03 (s, 2H), 1.54 (s, 56H), 1.33 (m, 36H), 1.24 (d,18H), 0.88 (d, 12H). The information on the molecular weight of CADPP: *M*_n_ = 22.6 k, *M*_w_ = 31.4 k, PDI = 1.39.

## 3. Results and Discussion

### 3.1. FT-IR Spectra

**Figure 1:**
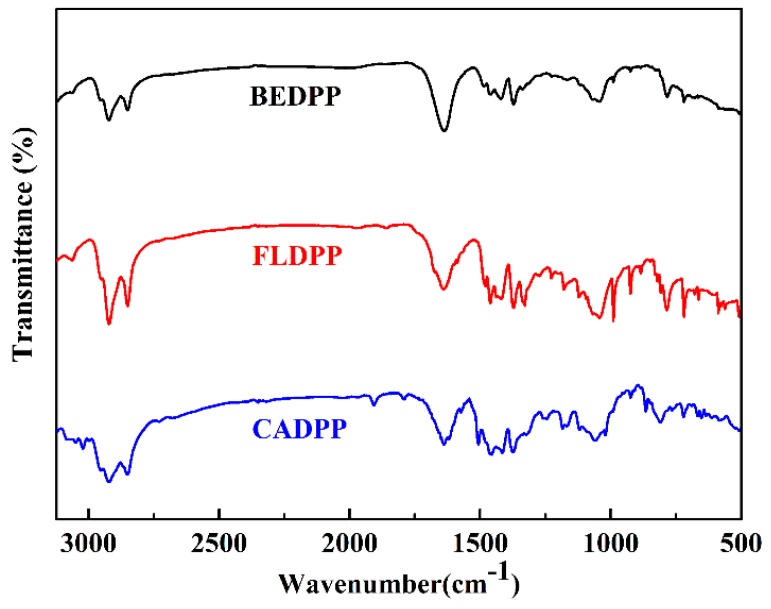
FT-IR spectra of BEDPP, FLDPP, and CADPP.

The infrared spectroscopy of these polymers is depicted in Figure 1. From the analysis of the infrared absorption spectra from the following diagram, we can determine that these polymers have similar absorption peaks, which is probably related to the same DPP acceptor unit, and the difference of donors may result in the changes of partial absorption peak intensity. Taking CADPP as an example, the absorption peak at 2930 cm^−1^ may be owing to the asymmetrical stretching vibration of the C–H bonds on the thiophene ring. The peak at 2850 cm^−1^ could be caused by the symmetrical stretching vibration of the saturated C–H bonds, the absorption peak at 1680 cm^−1^ was due to the skeleton vibration of the C=O bonds (acceptor), and the absorption peak at 1465 cm^−1^ maybe due to the skeleton vibration of the benzene ring(donor), the peak at 1443 cm^−1^ was probably the bending vibration of the C–H bonds on the methylene groups of the acceptor alkyl chains, the peak at 1362 cm^−1^ was possible the in-plane bending vibration of the C–H bonds on the methyl units. The peak at 1066 cm^−1^ could be the stretching vibration of C–N bonds on the DPP group or carbazole unit. In addition, the absorption peaks at 786 cm^−1^, 709 cm^−1^ may be caused by the out-of-plane bending vibration of the C–H bonds on the benzene rings or thiophene rings [38]. According to the detailed analysis of the characteristic absorption peaks, the backbones of CADPP included a DPP unit and a carbazole unit.

### 3.2. XPS Investigation of the Polymer

**Figure 2:**
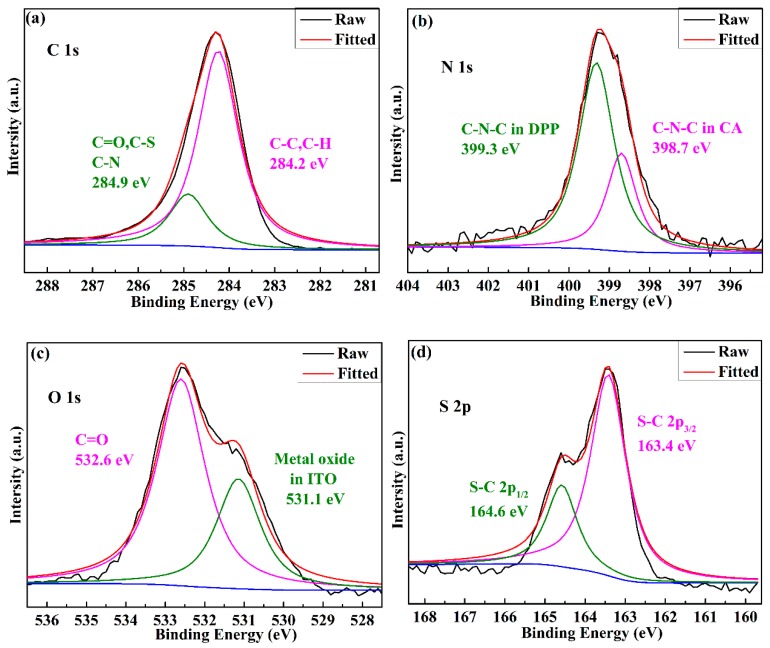
High-resolution XPS spectra of the CADPP thin films: (**a**) C 1s, (**b**) N 1s, (**c**) O 1s, and (**d**) S 2p. The raw (black) curve and the fitted (red) curve are shown in solid lines, respectively. The pink and green curves are just used to indicate the different individual peaks that constituted the fitted (red) peak.

In order to detect the elemental compositions of C, N, O, and S along the polymer backbones, the materials of BEDPP, FLDPP, and CADPP were characterized by X-ray photoelectron spectra (XPS) measurements. The results were recorded and shown in Figure 2 (Figure S4 and Figure S5 are shown in the supplementary material). Taking the polymer CADPP for example, the *C*1s raw peak of the polymer CADPP can be split into two peaks, as showed in Figure 2a. The peak located at 284.2 eV was attributed to the C atoms in C–C bonds and C–H bonds, which could be originated from the carbon atoms of the monomers including carbazole, thiophene and DPP blocks. The peak at 284.9 eV was assigned to the corresponding C atoms in C=O, C–S, C–N bonds of the two monomer units. The N 1s peak spectrum (shown in Figure 2b) can be disintegrated into two peaks, the peak at 398.7 eV may be attributed to the N atoms in C–N–C of carbazole block [39], and the peak at 399.3 eV was typical of the atom in C–N–C bond in DPP unit [40]. The corresponding filled areas of the two peaks in N 1s matched to the feed ratios of DPP and carbazole units, respectively. Figure 2c illustrates that two peaks of the O 1s profile, the peak at 532.6 eV was attributed to O atoms in C=O unit of DPP unit and the peak at 531.1 eV can be resulted in the metal oxide in ITO substrates. The S 2p spectra peaks (shown in Figure 2d) can be separated into two types of typical spin orbit peaks including S 2p^3/2^ (163.4 eV) and S 2p^1/2^ (164.6 eV), which were caused by the S atoms in C–S bond of the thiophene ring [38]. XPS curves of the CADPP showed that the polymer was synthesized successfully by incorporating the two monomers of carbazole and DPP units. Similar consequences were obtained from the polymers BEDPP (Figure S4) and FLDPP (Figure S5).

### 3.3. Electrochemical Characterization

**Figure 3:**
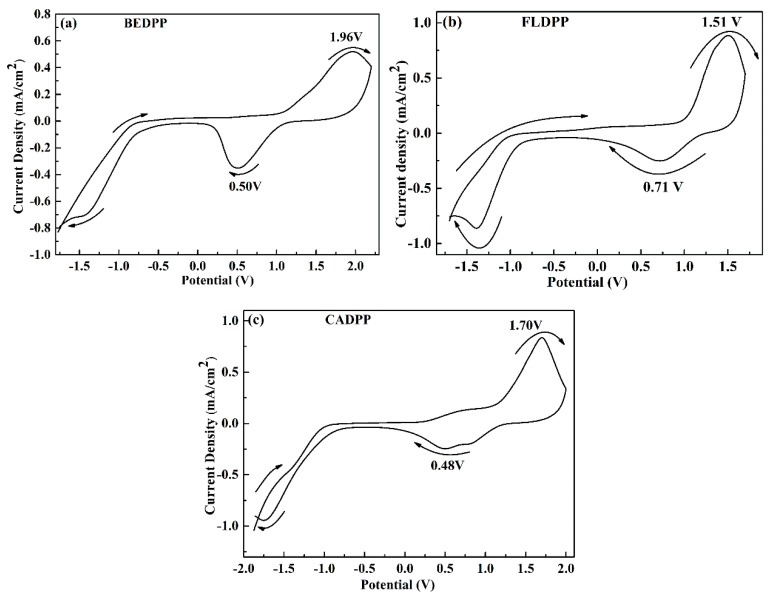
Cyclic voltammograms of spray-coated BEDPP (**a**), FLDPP (**b**), and CADPP (**c**) film. The direction of the arrow was based on the measurement procedure of the spray-coated indium tin oxide (ITO) films in 0.2 M TBAPF_6_ /ACN electrolyte solution at a scan rate of 100 mV/s.

Electrochemical property was determined by cyclic voltammetry (CV) experiment which was used to observe the oxidation reduction behavior and calculate the initial oxidation potential of the polymer film. The polymers were dissolved in chloroform completely, and sprayed uniformly on the ITO-coated glass with a spray gun. The voltage scanning range of BEDPP, FLDPP, and CADPP were −2~2 V with the scanning rate being the 100 mV/s, and the supporting electrolyte was 0.2 M TBAPF_6_ (dissolved in acetonitrile). After one round of scanning, the CV curves of the three polymers were obtained and shown in Figure 3. It can be found that there are a pair of obvious redox peaks in every polymer at positive potential, and the redox peaks at1.96 V/0.50 V, 1.51 V/0.71 V, and 1.70V/0.48 V for BEDPP, FLDPP, and CADPP, respectively, which proved that all three polymers having obvious p-type doping characteristics. During the process of negative potential scanning, all three polymers exhibited irreversible n-type doping, and the onset oxidation potentials (*E*_onset_) calculated were 1.05 V for BEDPP, 0.95 V for FLDPP, and 0.93 V for CADPP, indicating the declining trend of the *E*_onset_ with the ability of donating-electrons strengthening. A classical explanation was that the strong donor units will contribute to improve the HOMO level, reduce the required potential and make the radical cation more susceptible to oxidation [19]. The results showed that the alternative of donor units can modify the redox properties of the D-A type conjugated polymer effectively. In addition, E_HOMO_ value of three polymers can be calculated by the equation below [32]:

*E*_HOMO_ = −(4.8 + (*E*_onset_ − 0.55))eV (1)

We can obtain the highest occupied molecular orbital energy (*E*_HOMO_) level of BEDPP, FLDPP, and CADPP were −5.30, −5.20 and −5.18 eV, respectively.

### 3.4. Optical Properties

**Figure 4:**
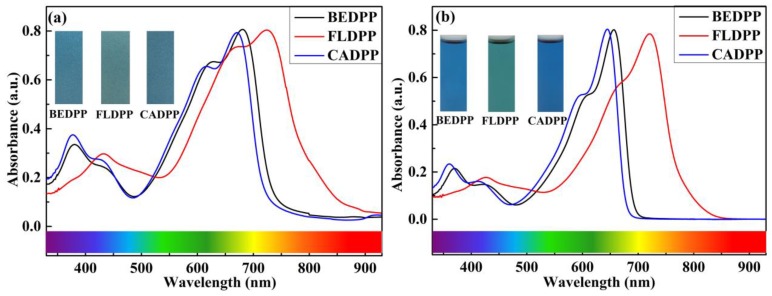
UV-VIS absorption spectra of BEDPP, FLDPP, and CADPP films in TBAPF_6_/CAN (**a**) in the neutral state and those dissolved in CHCl_3_ (**b**).

The optical properties of the three films and solutions for BEDPP, FLDPP and CADPP were detected in the neutral state by using UV–VIS–NIR absorption spectroscopy. The details related to the optical performances of the three polymers and some other polymers with similar structures are summarized in Table 1, and the corresponding optical behaviors of three polymers are shown in Figure 4. It was easy to recognize that three kinds of polymers in the visible light region having two obvious absorption peaks in Figure 4, the emergence of the two absorption peaks attributed to π-π* transition and intramolecular charge transformation, and this is the unique characteristics of D-A type conjugated polymer [38]. The maximum absorption peaks of the polymer films for BEDPP, FLDPP, and CADPP are located at 684, 724, and 675 nm, respectively, while the maximum absorption peaks of three polymer solutions are 656, 720, and 645 nm, respectively. In addition, we found that, compared with its solution for the same polymers, the maximum absorption peaks of the films showed an obvious red-shift. This phenomenon could result in the aggregation effect in the solid-state, because the π-π* stacking effect in the polymer films enhanced the molecule interaction and reduced the absorption energy [41]. Compared with the polymer solutions, the films have no obvious color diversification, BEDPP exhibited sky blue, FLDPP revealed cyan, and CADPP showed pure blue.

Through the ultraviolet image of the films, the onset absorption wavelength (*λ*_onset_) of the three polymers of BEDPP, FLDPP, and CADPP were 801, 735, and 720 nm, respectively. According to the following Equations [25,32]:

*E*_g_ = 1240/*λ*_onset_ (2)

*E*_HOMO_= −(4.8 + (*E*_onset_ − 0.55))eV (3)

*E*_LUMO_ = *E*_HOMO_ + *E*_g_ (4)

The number 0.55 in the formula is a correction parameter, which was used to calibrate the Ag pseudo-reference electrode to the ferrocenium/ferrocene couple(Fc/Fc+). The optical band-gap energy (*E*_g_) of three polymers are calculated to be the 1.55, 1.69, and 1.72 eV (based on the equations of *E*_g_ = 1240/*λ*_onset_), respectively. This may be the highly matching degree between the benzene ring and the DPP structure of BEDPP, which is more beneficial to electronic transmission and generated the low band gap.

### 3.5. Quantitative Calculation

**Figure 5:**
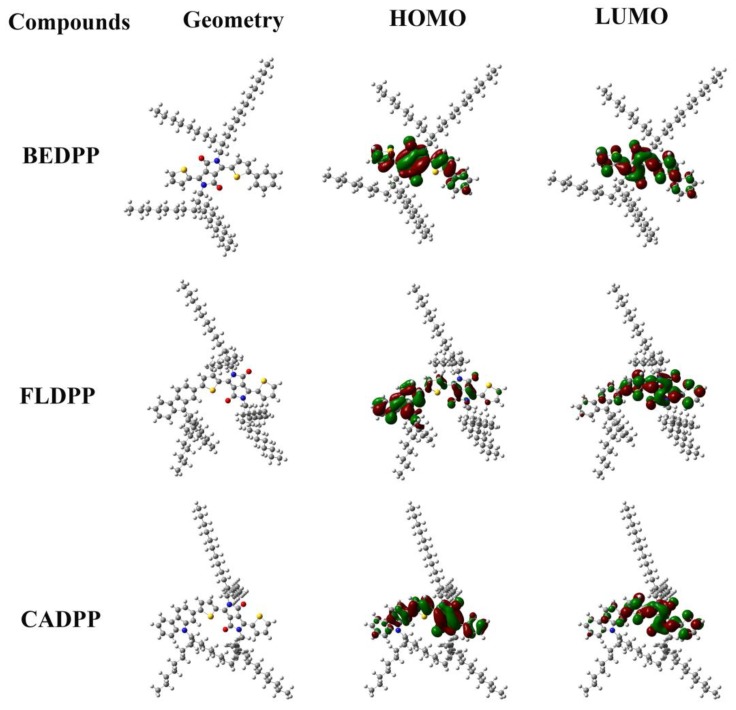
Molecular orbital diagram of HOMO and LUMO of BEDPP, FLDPP, and CADPP.

Quantitative calculation can be used to analyze the space structure, electron distribution, and theoretical band gap value of the polymers from the theoretical perspective. Gaussian 09 software (Shanghai eMolecular Technology Inc., Ltd, Shanghai, China) was used to conduct quantitative calculation based on density functional theory. The electron density distribution of HOMO and LUMO of the materials can be seen from Figure 5. The HOMO and LUMO orbitals were primarily delocalized on the aromatic rings and rarely involved the alkyl groups, which indicated that each material having the excellent planar conjugated structures. For BEDPP, the angle between the benzene ring (donor) and thiophene was 2.23°, and the angle of thiophene and DPP unit was1.49°, owing to the great planar structure. For FLDPP, the electrons of the HOMO level were mainly concentrated on the fluorene ring that resulted with common planar behavior, so it hindered the electrons transmission and limited the HOMO-level electrons’ delocalization. The introduction of the fluorene ring makes the angle increase to 4.69° between the DPP unit and fluorene ring, and the angle of thiophene and DPP unit was 2.56°. For CADPP, the introduction of nitrogen atoms makes carbazole have a stronger electron-donating effect. Meanwhile, the angle was 2.09° between thiophene and the carbazole unit, with the thiophene and DPP unit being 6.02°. After quantitative calculation, the band-gap energy values of quantitative calculation (*E*_g,cal_) were 2.09, 2.18, and 2.25 eV for BEDPP, FLDPP, and CADPP, respectively, which were obviously higher than those of the actual tested band gap values (*E*_g,op_) according to the optical properties of the films (1.55, 1.69, and 1.72 eV for BEDPP, FLDPP, and CADPP). The reason for this phenomenon may be the reaction polymerization degree and solvent effect on the experiment [17]. Based on the database above, whether *E*_g,op_ or *E*_g,cal_ showed the same sequence followed as CADPP > FLDPP > BEDPP, this demonstrated that the electron-donating capacity and matching degree of donor-acceptor having the important effects for the band gap value of the polymers.

### 3.6. Spectroelectrochemistry

**Figure 6:**
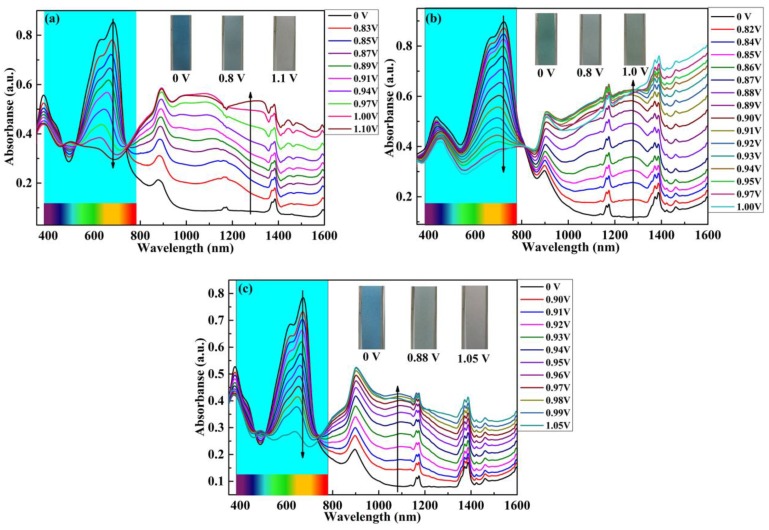
Spectroelectrochemistry of BEDPP (**a**), FLDPP (**b**) and CADPP (**c**) and their photos in neutral states and oxidized states.

Spectroelectrochemistry is usually used to record the variation of the absorption spectrum of the films ranging from 300 to 1600 nm with the different applied voltages. The spectrum of the three polymer films at progressively increased voltages from 0 V (neutral state) to 1.10 V (oxidized state) was recorded in Figure 6. For BEDPP (shown in Figure 6a), there have two absorption peaks at 380 and 684 nm in the visible region (neutral state). The first peak at 380 nm with a narrow absorption region from 330 to 460 nm, the second peak at 684 nm revealed a wide absorption band from 518 to 740 nm, which could result with the π-π* transitions and intramolecular charge transformation effects. Interestingly, the absorption valley at 489 nm allowed the transmittance of blue light, which makes BEDPP generate a sky blue color. When the voltages increased from the neutral state (0 V) to the fully oxidized state (1.10 V), the intensity of two peaks in the visible light region weakened gradually while the intensity in the near-infrared region (1100 nm) strengthened gradually. The probable reasons may be the reduction of the π-π* transformation electrons and the formation of polaron and bipolaron, which can promote the rearrangement of the electrons of the polymers [20]. Apparently, the colors of the polymer BEDPP film changes from sky blue (0 V) to light brown (1.10 V), showing that the color variations were significant and attractive.

For FLDPP (shown in Figure 6b), it can be seen that the visible light region was divided into two parts with the voltage increasing continuously (0–1.0 V). The two absorption peaks at 436 nm and 724 nm in the visible region (neutral state) with the absorption valley at 552 nm. Meanwhile, the new absorption band in the near-infrared region (1180 nm) indicated the formation of a polaron and a bipolaron [43]. The color of FLDPP was changed from cyan color in the neutral state (0 V) to transparent gray color (1.0 V) in the oxidized state. Similarly, for CADPP (shown in Figure 6c), the two π-π* transition peaks were located at 377 and 675 nm in the neutral state. The spectral shifts to the near-infrared region due to the doping process, at the same time, the generation of the polaron caused a new absorption peak at 903 nm. Due to the weakened of the high-energy absorption peaks in the visible region and the strengthened of the low-energy absorption peaks in the near-infrared region, the polymers demonstrated the corresponding color variations. Therefore, CADPP displays a pure blue color in the neutral state (0 V) and a light gray color in the oxidized state (1.05 V). The three polymers have obvious color diversification performances, which will be beneficial to the developments of electrochromic materials.

All three polymers showed unexpected and irregular small peaks at about 1174 and 1388 nm, and these peaks can be regarded as interfering peaks, which might be caused by the performance of the UV–VIS–NIR spectrophotometer itself and not related with the optical properties of the polymers.

### 3.7. Electrochromic Switching

**Figure 7:**
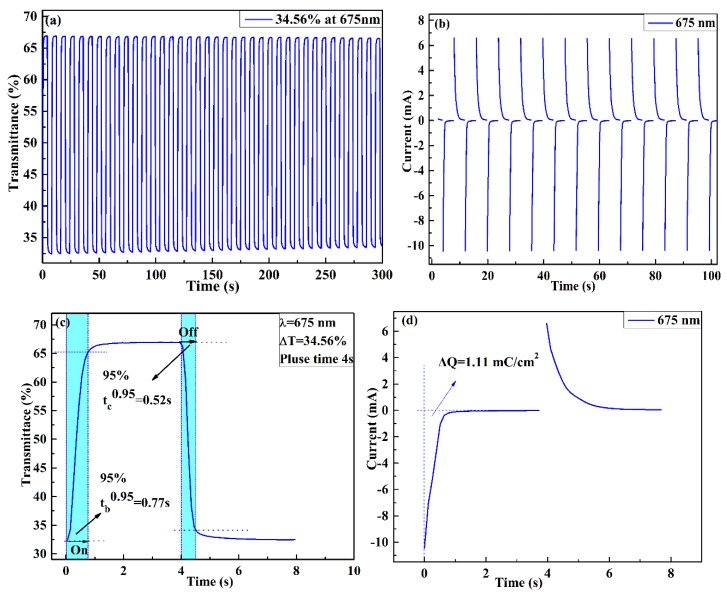
(**a**) Transmittance-time curve of CADPP lasts for 300 s at 675 nm. (**b**) Current-time switching curve of CADPP film between 0 V and 1.05 V in a time interval of 4 s. (**c**) The bleaching time (*t*_b_) and the coloration time (*t*_c_) of CADPP at 675 nm. (**d**) The second cycle of the current-time curve.

Electrochromic switching is the principal method to investigate the application values of conjugated polymers including the factors of response time (t_95%_), coloration efficiency (η), and cycling stability. The parameters of the polymers were recorded by the method of multi-potential steps employing the three-electrode systems. The whole process requires a spectrophotometer and electrochemical workstation to monitor the performance of the films at different voltages (from 0 V to the oxidized states). The polymer films were switched using potential square waves in definite interval times and cycle times in the dynamic studies. For the polymer CADPP (Figure 7), the optical contrasts of the film were 34.56% at 675 nm by switching the potential from the 0 to 1.05 V with an interval time of 4 s (shown in Figure 7a). The current-time curve was recorded and revealed a stable intensity in 100 s (shown in Figure 7b). Response time refers to the necessary time for polymers to achieve 95% of the optical contrasts between colored and bleached processes. Hence, the bleaching time (*t*_b_) and the coloration time (*t*_c_) was calculated to be 0.77 and 0.52 s (shown in Figure 7c), respectively, which demonstrated that the polymers possess great charge transfer performance. The consumed charge density of CADPP (1.11 mC/cm^2^) at 675 nm can be calculated according to the second cycle current intensity parameter (shown in Figure 7d).

**Figure 8:**
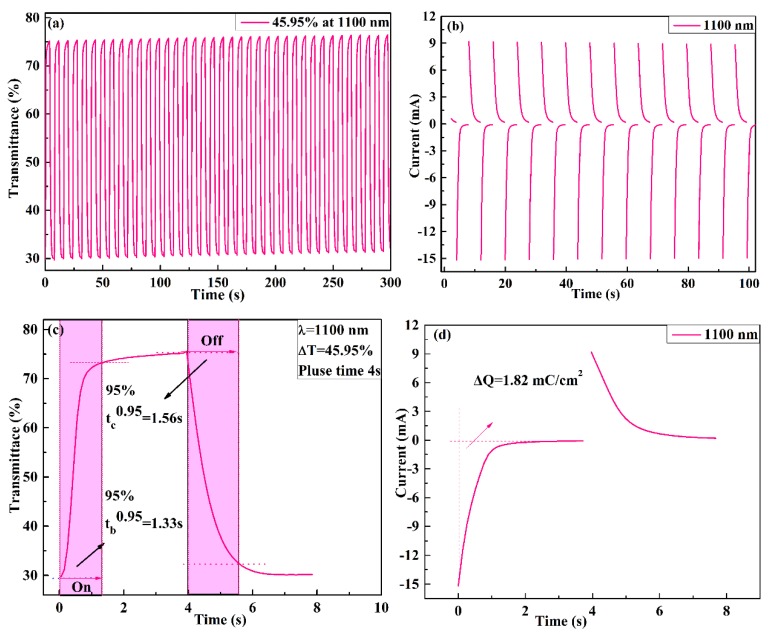
(**a**) Transmittance-time curve of CADPP lasts for 300 s at 1100 nm. (**b**) Current-time switching curve of CADPP film between 0 V and 1.05 V in a time interval of 4 s. (**c**) The bleaching time (*t*_b_) and the coloration time (*t*_c_) of CADPP at 1100 nm. (**d**) The second cycle of the current-time curve.

With an interval time of 4 s, the relevant kinetic switching databases of 1100 nm (near IR region) for polymer CADPP were summarized as follows (Figure 8). The optical contrasts of the films were 45.95% at 1100 nm by switching the potential from the 0 to 1.05 V (shown in Figure 8a). The current-time curve was recorded and the intensity of the current was kept stable for 100 s (shown in Figure 8b). The bleaching time (*t*_b_) and the coloration time (*t*_c_) were 1.33 s and 1.56 s (shown in Figure 8c), respectively, which demonstrated that the polymers consumed more time to change color in the near-IR region compared with 675 nm, possibly due to the formation of a polaron limiting the speed of the ion transport performance. The consumed charge density of CADPP (1.82 mC/cm^2^) at 1100 nm can be calculated from the current intensity based on the second cycle parameter (shown in Figure 8d).

The detailed electrochromic switching performances of BEDPP and FLDPP are shown in Figure S6 (684 nm for BEDPP), Figure S7 (1100 nm for BEDPP), Figure S8 (724 nm for FLDPP), and Figure S9 (1280 nm for FLDPP), respectively. The corresponding data of BEDPP and FLDPP were summarized in Table 2. From the Table 2, we found that the optical contrasts of the BEDPP film were 24.95% at 684 nm (shown in Figure S6a) and 41.32% at 1100 nm (shown in Figure S7a), meanwhile, the optical contrasts of the FLDPP film were 29.24% at 724 nm (shown in Figure S8a) and 42.39% at 1280 nm (shown in Figure S9a). For BEDPP, the response time is 1.21 s for *t*_b_ and 0.66 s for *t*_c_ at 684 nm (shown in Figure S6c), 1.98 s for *t*_b_ and 0.96 s for *t*_c_ at 1100 nm (shown in Figure S7c), and for FLDPP, the response time is 0.61 s for *t*_b_ and 0.52 s for *t*_c_ at 724 nm (shown in Figure S8c), 1.49 s for *t*_b_ and 1.66 s for *t*_c_ at 1280 nm (shown in Figure S9c). To sum up, three polymers possess satisfactory response times. The planar structure accelerates the speed of the doping and de-doping process and makes the polymers have fast response times. The comparison demonstrated that the response time of CADPP was significantly better than BDDPP and FLDPP, which may be related to the carbazole group having a stronger electron donor capacity.

The coloration efficiency (CE, *η*) is also an important parameter for applications of the electrochromic materials. It is defined that the optical density (Δ*OD*) for the charge consumed per unit electrode area (Δ*Q*), and Δ*Q* represents the consumed charges (*Q*) in the per unit electrode area (*A*) during the process of injected or ejected electrons [42]. The corresponding equations are given below:

Δ*OD* = lg (*T*_b_/*T*_c_) (5)

Δ*Q* = *Q*/*A* (6)

*η* = Δ*OD*/Δ*Q* (7)

T_b_ and T_c_ are the transmittances before and after coloration, respectively, and *η* denotes the coloration efficiency (CE). Coloring efficiency is a ratio of optical density change and charge density, which is related to the energy consumption of electrochromic materials. The higher coloring efficiency means the lower energy consumption. It was calculated that the coloring efficiency of BEDPP was 195.74 cm^2^·C^−1^ at 684 nm and 248.63 cm^2^·C^−1^ at 1100 nm. The coloring efficiency of FLDPP at 724 nm was 249.06 cm^2^·C^−1^ and 197.31 cm^2^·C^−1^ at 1280 nm. CADPP has the highest coloring efficiency of 288.42 cm^2^·C^−1^ at 675 nm. The data above revealed that the CADPP has the best comprehensive performances compared to BEDPP and FLDPP.

**Figure 9:**
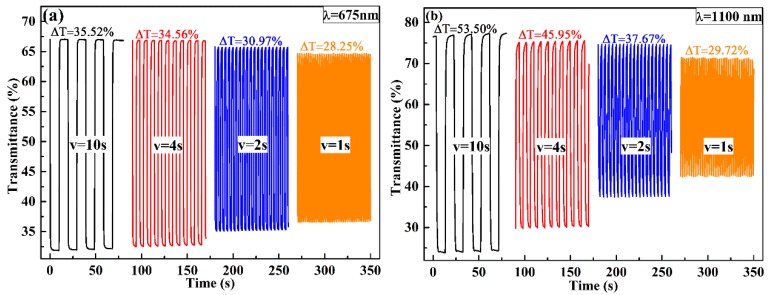
Electrochromic switching of CADPP at 675 nm (**a**) and 1100 nm (**b**).

It is important to the study the dependence of the optical contrasts on the retention times of the polymers under the course of the dynamic switching. For this objective, the interval times (from the neutral to oxidized state) were set at 10 s, 4 s, 2 s, and 1 s, respectively, to monitor the optical contrasts. For CADPP, the optical contrasts at 675 nm were recorded as 35.52%, 34.56%, 30.97%, and 28.25%, respectively, at intervals of 10 s, 4 s, 2 s, and 1 s, respectively (Figure 9a). The optical contrasts were reduced by 20.5% with the time interval being reduced from 10 s to 1 s. At a wavelength of 1100 nm, the transmittance contrasts were 53.50%, 45.95%, 37.67%, and 29.72% at intervals of 10 s, 4 s, 2 s, and 1 s, respectively (Figure 9b). The optical contrasts reduction of 44.4% was calculated as the time interval from 10 s to 1 s. By comparing the response time of CADPP the less decrease of the optical contrasts with the faster response times in the dynamic switching studies can be determined.

For BEDPP, the optical contrasts at 684 nm were recorded as 26.12%, 24.98%, 24.06%, and 20.46%, respectively, at intervals of 10, 4, 2, and 1 s (shown in Figure S10a). As the time interval was reduced from 10 s to 1 s, the optical contrast was reduced by 21.6%. At a wavelength of 1100 nm, the transmittance contrasts were 50.03%, 41.32%, 29.38%, and 16.86% at intervals of 10, 4, 2, and 1 s, respectively (shown in Figure S10b). For FLDPP, the optical contrasts at 724 nm were recorded as 30.39%, 29.24%, 23.64%, and 20.01%, respectively, at intervals of 10, 4, 2, and 1 s (shown in Figure S11a). As the time interval was reduced from 10 s to 1 s, the optical contrast was reduced by 34.1%. At a wavelength of 1280 nm, the transmittance contrasts were 45.12%, 42.39%, 34.72%, and 25.58% at intervals of 10, 4, 2 and 1 s, respectively (shown in Figure S11b). It can be observed that the three polymers showed good kinetic stability, which can be expected from potential candidate materials for electrochromic devices.

### 3.8. Colorimetry

**Figure 10:**
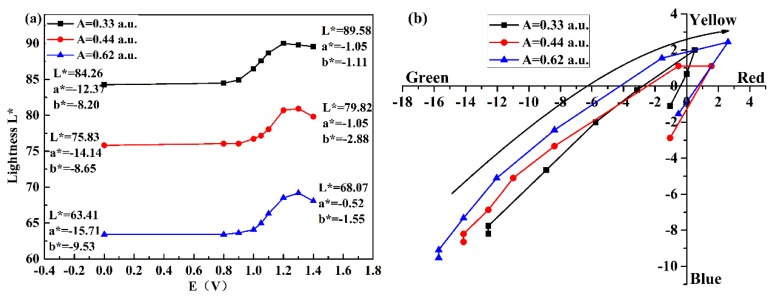
Lightness variation coordinates (**a**) and a* − b* values color space (**b**) of CADPP during p-doping processing from neutral state to oxidized states based on three different thickness films. The direction of the arrows in (**b**) indicates the color changing trends of the polymer films.

Colorimetry is a quantitative method to describe the color changes and brightness variations of the polymers during the redox processing. Here, the CIE 1976 L*a*b* color space was adopted to test the color of polymers at different potentials, L* signifies the lightness from black (0) to white (100), a* denotes the contrast between red and green, and b* means the contrast between yellow and blue [44]. Three different thickness films were sprayed on ITO glass with 0.2 M TBAPF_6_/CAN as the electrolyte. As shown in Figure 10, for CADPP, the maximum optical absorption of three films are 0.33 a.u, 0.44 a.u, and 0.62 a.u, respectively. When the maximum absorbance value of the film is 0.33 a.u, L*, a*, and b* are 84.26, −12.37 and −8.20 at 0 V, the L*, a*, and b* are 89.58, −1.05 and 1.11 at 1.40 V, respectively. Moreover, the films begin to be oxidized with the lightness increasing when the potential up to the *E*_onset_ (0.93 V), and the lightness of the three films are 89.58, 79.82, and 68.07, respectively, with the films being oxidized completely. It is a typical feature of cathodically-coloring polymers that the lightness of the film was improved obviously from neutral to oxidized state. In addition, according to a* − b* graph, the color change trends of polymer films can also be intuitively observed, and the similar starting point could explain the phenomenon of the blue color in the neutral state. From the neutral state to the oxidation state, the color of CADPP changes from the third quadrant to the second quadrant and the first quadrant to the third quadrant. BEDPP changes from the third quadrant to the second quadrant, while the FLDPP changes from the third quadrant to the first quadrant. Based on this, it can be seen that the color changes of CADPP were more abundant than that of BEDPP and FLDPP. The colorimetry details of BEDPP (Figure S12) and FLDPP (Figure S13) are presented in the supplementary material.

**Figure 11:**
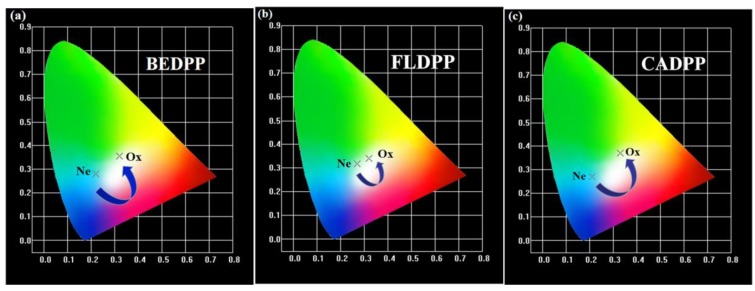
The color space plots for BEDPP (**a**), FLDPP (**b**), and CADPP (**c**) at the oxidation state (Ox) and neutral state (Ne).

The color space plots could indicate the color changes visually for the three polymers, which are depicted in Figure 11. We determined that the three materials exhibited a blue color in their neutral states from the figure below. As the applied potential increased, the color of every polymer moves toward to the white region in the center of the color space plot. Interestingly, the three color states can be easily compared by drawing the linear distance between the neutral point and the oxidized point. Since the position on the color space plot represents different colors, the distances from one coordinate point to another can be explained as the color difference between the two potentials denoted by the coordinate points [45]. Therefore, the color difference of CADPP just matches the variation trend of a* − b* graph, which indicates that the CADPP polymer possesses abundant color changes.

### 3.9. Thermogravimetric Analysis

**Figure 12:**
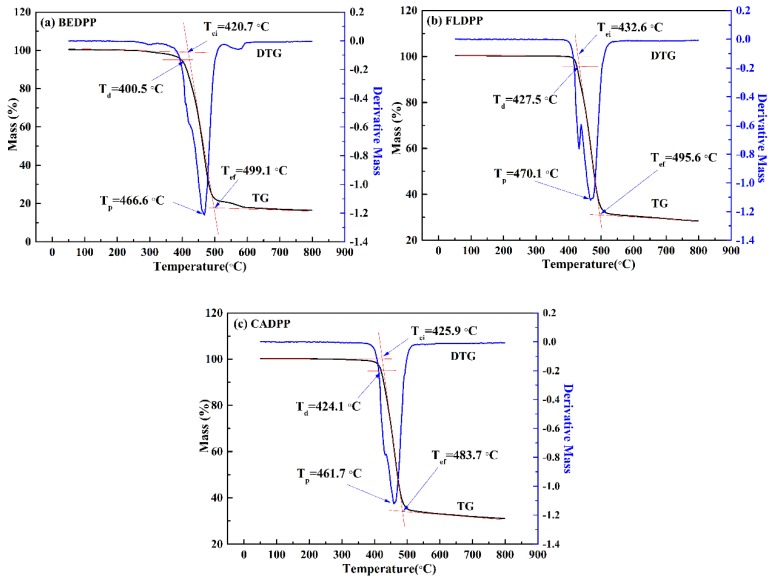
Thermogravimetric and derivative thermogravimrtric analysis curves of the polymers BEDPP (**a**), FLDPP (**b**), and CADPP (**c**).

Thermal stability is directly related to the application value of electrochromic materials. Thermogravimetric analysis (TG) is an effective method to characterize the thermal stability of polymers. The derivative thermogravimetric analysis (DTG) is the derivative of the TG curve based on temperature variety, which indicatesthe mass change rate following time. From the TG curves we can determine that the three polymers’ initial decomposition temperatures *T*_ei_ were 420.7 °C (BEDPP), 432.6 °C (FLDPP), and 425.9 °C (CADPP) (Figure 12). The final decomposition temperatures *T*_ef_ were 499.1 °C, 495.6 °C, and 483.7 °C, for BEDPP, FLDPP, and CADPP, respectively. The initial decomposition temperature T_d_ was 400.5 °C for BEDPP, 427.5 °C for FLDPP, and 424.1 °C for CADPP. The prestissimo decomposition temperature *T*_p_ was 466.6 °C for BEDPP, 470.1 °C for FLDPP, and 461.7 °C for CADPP (Figure 12). The char yields of the polymers were 18.2% for BEDPP, 29.8% for FLDPP, and 31.2% for CADPP at the final 800 °C (Figure 12). The related data were summarized in Table 3. In summary, all three polymers have satisfactory thermal stability and can meet the requirements based on the environmental temperature for various EC devices.

## 4. Conclusions

In this study, three new-fashioned D-A type conjugated polymers, BEDPP, FLDPP, and CADPP, were successfully synthesized based on different donor units of benzene, fluorine, and carbazole. The multifunctional electron deficient molecule diketopyrrolopyrrole (DPP) was introduced and employed as the acceptor unit. The characterization methods of NMR, IR, and XPS were carried out to confirm the structure of the polymers. The photoelectric properties of the three polymers were then characterized in detail. The narrow optical band gaps of BEDPP, FLDPP, and CADPP were 1.55, 1.69, and 1.72 eV, respectively. It was found that BEDPP and CADPP showed a stable saturated blue color in neutral state while FLDPP displayed a cyan color. In addition, FLDPP (0.61 s for *t*_b_ and 0.52 s for *t*_c_) and CADPP (0.77 s for *t*_b_ and 0.52 s for *t*_c_) revealed fast response times in the visible region that are comparable to that of the other D-A type DPP-containing polymers. CADPP also illustrated outstanding coloration efficiency of 288.42 cm^2^·C^−1^ and satisfactory optical contrasts of 34.56% at 675 nm. In terms of thermogravimetric stability, the three polymers maintain good stability at 400 °C, which can satisfy the temperature requirements of the EC device. Compared with the performances of the three polymers, we found that CADPP has the best electrochromic behavior. This may be related to the fact that the carbazole unit has the stronger electron-donating capacity, and the planar structure is also crucial to the electrochromic performance. In summary, the attractive property of the three polymers demonstrated that they deserved further study, especially in the fields of neutral blue (RGB) and thermal stability.

## Figures and Tables

**Table 1:** The parameters related to the optical absorption and band gaps of BEDPP, FLDPP, CADPP, and some similar polymers.

Copolymers	*λ*_onset_(Film)	*λ*_max_(Solution)	^1^*λ*_max_(Film)	*E*_onset_	*E*_g_	HOMO	LUMO
	nm	nm	nm	V	eV	eV	eV
BEDPP	801	656	684	1.06	1.55	−5.30	−3.76
FLDPP	735	720	724	0.95	1.69	−5.20	−3.51
CADPP	720	645	675	0.93	1.72	−5.18	−3.46
PD-1 ^a^	798	757	725	0.89	1.55	−5.73	−4.18
PD-2 ^a^	817	787	732	0.92	1.52	−5.76	−4.24
POOC ^b^	713	561	582	1.04	1.74	−5.46	−3.72
POOF ^b^	713	562	579	1.13	1.74	−5.55	−3.81
LGC-D055 ^c^	849	650,695	695,767	0.86	1.46	−5.11	−3.66

^1^*λ*_max_ signifies the wavelength corresponding to the maximum absorption peak in the absorption spectra. ^a^ data from reference [42]. ^b^ data from reference [33]. ^c^ data from reference [26].

**Table 2:** Optical contrast (Δ*T*%), response time (*t*_95%_) and coloration efficiency (*η*) of BEDPP, FLDPP, CADPP, and other polymers with similar structures.

Copolymers	*λ*	Δ*T*%	Response Time (*t*_95%_)		Coloration Efficiency (*η*)
	nm	%	*T*_b_(s)	*T*_c_(s)	cm^2^·C^−1^
BEDPP	684	24.95	1.21	0.66	195.74
	1100	41.32	1.98	0.96	248.63
FLDPP	724	29.24	0.61	0.52	249.06
	1280	42.39	1.49	1.66	197.31
CADPP	675	34.56	0.77	0.52	288.42
	1100	45.95	1.33	1.56	227.09
PD-2 ^a^	732	29.76	3.04	0.47	220.18
	1620	67.74	0.94	3.20	267.95
P(NO2-3Cz) ^b^	432	25	3.81	1.13	62
	710	52	4.23	1.06	35

^a^ data from reference [42]. ^b^ data from reference [36].

**Table 3:** TG, DTG data for BEDPP, FLDPP, and CADPP.

Compound	^a^*T*_ei_(°C)	^b^*T*_ef_(°C)	^c^*T*_p_(°C)	^d^*T*_d_(°C)	^e^% Char
BEDPP	420.7	499.1	466.6	400.5	18.2
FLDPP	432.6	495.6	470.1	427.5	29.8
CADPP	425.9	483.7	461.7	424.1	31.2

^a^*T*_ei_ signifies the intersection of the tangent of the baseline and the tangent line of center curve of the TG curve. ^b^*T*_ef_ refers to the point at the intersection point of the tangent line of the center and the end section of the TG curve. ^c^*T*_p_ denotes to the inflection point that can be drawn from the lowest point of DTG curve. ^d^*T*_d_ means the point at which a 5% weight decrease of the material occurs. ^e^% char represents the percentage of the residual carbon ash to the quality of the initial samples at the final temperature [41].

## Supplementary Materials

The following are available online at https://www.mdpi.com/2073-4360/11/12/2023/s1. Figure S1: ^1^H NMR spectra (a) and ^13^C NMR (**b**) of the compound B; Figure S2: ^1^H NMR spectra (a) and ^13^C NMR (b) of the monomer C; Figure S3: ^1^H NMR spectrum of BEDPP (a), FLDPP (b) and CADPP (c); Figure S4: XPS spectra of polymer BEDPP: (a) *C*1s, (b) N1s, (c) O1s, and (d) S2p; Figure S5: XPS spectra of polymer FLDPP (a) C1s, (b) N1s, (c) O1s, and (d) S2p; Figure S6: (a) The transmittance-time curve of BEDPP lasts for 300 s at 684 nm. (b)The current-time switching curve of BEDPP film between 0 V and 1.10 V in a time interval of 4 s. (c) The bleaching time (*t*_b_) and the coloration time (*t*_c_) of BEDPP at 684 nm. (d) The second cycle of the current-time curve; Figure S7: (a) The transmittance-time curve of BEDPP lasts for 300 s at 1100 nm. (b) The current-time switching curve of BEDPP film between 0 V and 1.10 V in a time interval of 4 s. (c) The bleaching time (*t*_b_) and the coloration time (*t*_c_) of BEDPP at 1100 nm. (d) The second cycle of the current-time curve; Figure S8: (a) The transmittance-time curve of FLDPP lasts for 300 s at 724 nm. (b) The current-time switching curve of FLDPP film between 0 V and 1.0 V in a time interval of 4 s. (c) The bleaching time (*t*_b_) and the coloration time (*t*_c_) of FLDPP at 724 nm. (d) The second cycle of the current-time curve; Figure S9: (a) The transmittance-time curve of FLDPP lasts for 300 s at 1280 nm. (b) The current-time switching curve of FLDPP film between 0 V and 1.0 V in a time interval of 4 s. (c) The bleaching time (*t*_b_) and the coloration time (*t*_c_) of FLDPP at 1280 nm. (d) The second cycle of the current-time curve; Figure S10: Electrochromic switching of BEDPP at 684 nm (a) and 1100 nm (b); Figure S11: Electrochromic switching of FLDPP at 724 nm (a) and 1280 nm (b); Figure S12: Lightness variation coordinates (a) and a* − b* values color space (b) of BEDPP during p-doping processing from neutral state to oxidized states based on three different thickness films. The direction of the arrows in (b) indicates the color changing trends of the polymer films; Figure S13: Lightness variation coordinates (a) and a* − b* values color space (b) of FLDPP during p-doping processing from neutral state to oxidized states based on three different thickness films. The direction of the arrows in (b) indicates the color changing trends of the polymer films.
