# Supplementary material for: Synthesis and Characterization of Novel D-A Type Neutral Blue Electrochromic Polymers Containing Pyrrole[3-c]Pyrrole-1,4-Diketone as the Acceptor Units and the Aromatics Donor Units with Different Planar Structures
Source: Polymers (Basel). 2019 Dec 6;11(12):2023. doi: 10.3390/polym11122023 (PMC6960932; doi:10.3390/polym11122023)
Supplement: Supplementary file 1 [file polymers-11-02023-s001.pdf]

polymers

Supporting Information

Synthesis and Characterization of Novel D-A Type Neutral Blue Electrochromic Polymers Containing Pyrrole[3-c]Pyrrole-1,4-Diketone as the Acceptor Units and the Aromatics Donor Units with Different Planar Structures

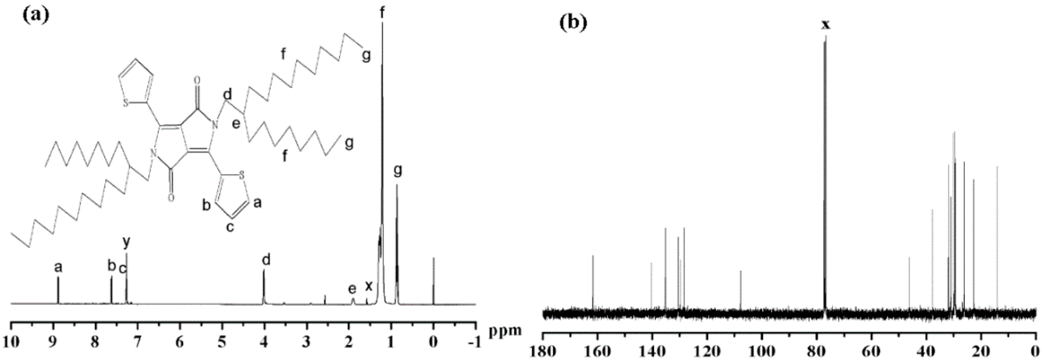

Figure S1. ^{1}H NMR (**a**) and ^{13}C NMR (**b**) spectra of compound B.

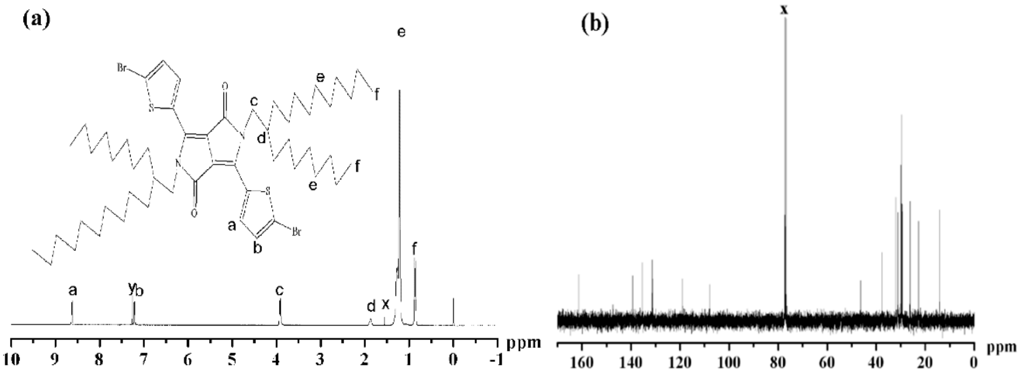

Figure S2. ^{1}H NMR (**a**) and ^{13}C NMR (**b**) spectra of monomer C.

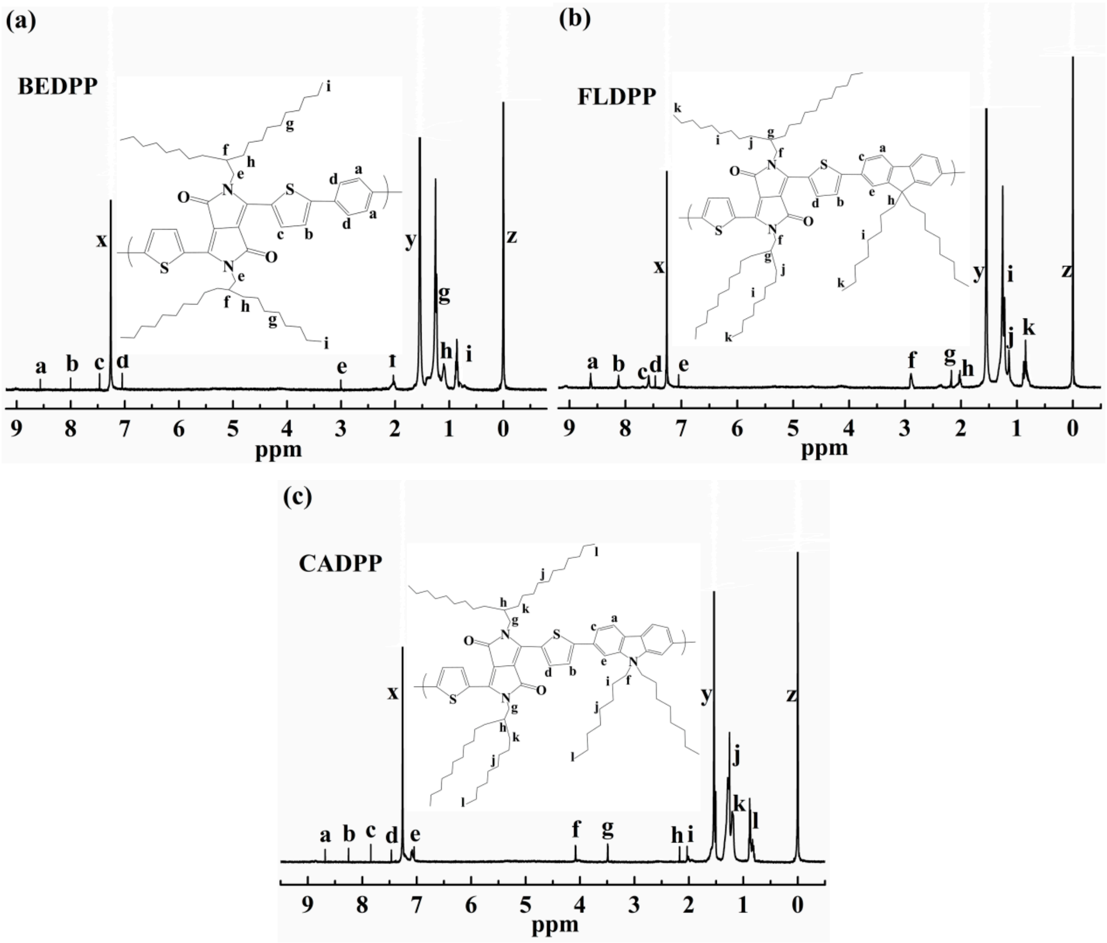

Figure S3. NMR spectrum of BEDPP(**a**), FLDPP(**b**), CADPP(**c**), $CDCl_3$ Solvent (7.26), water (1.54) and tetramethylsilane peaks (0) were marked by x, y, z, respectively.

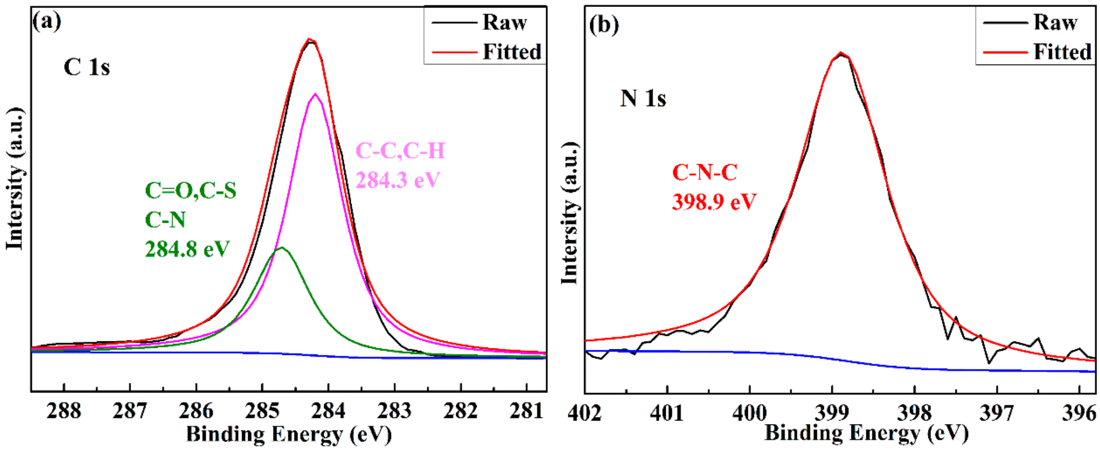

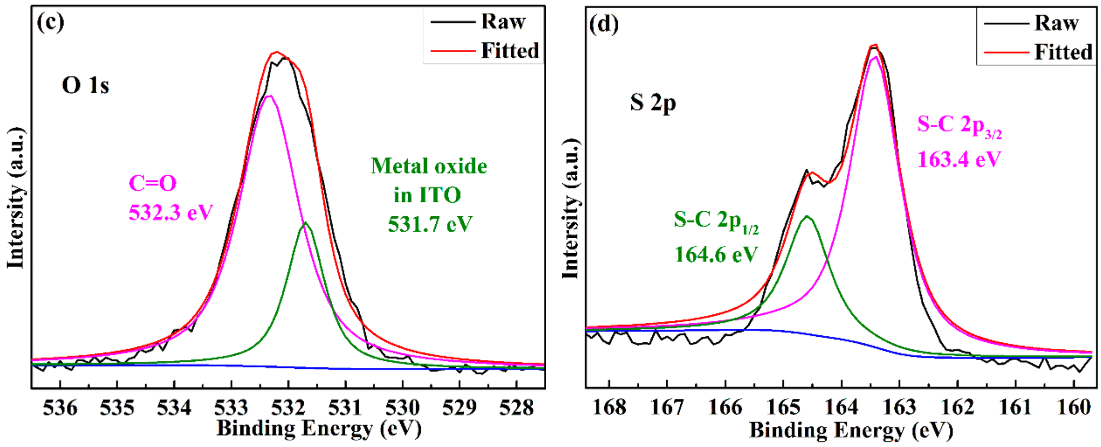

Figure S4. XPS spectra of polymer BEDPP: (**a**) C1s, (**b**) N1s, (**c**) O1s and (**d**) S2p.

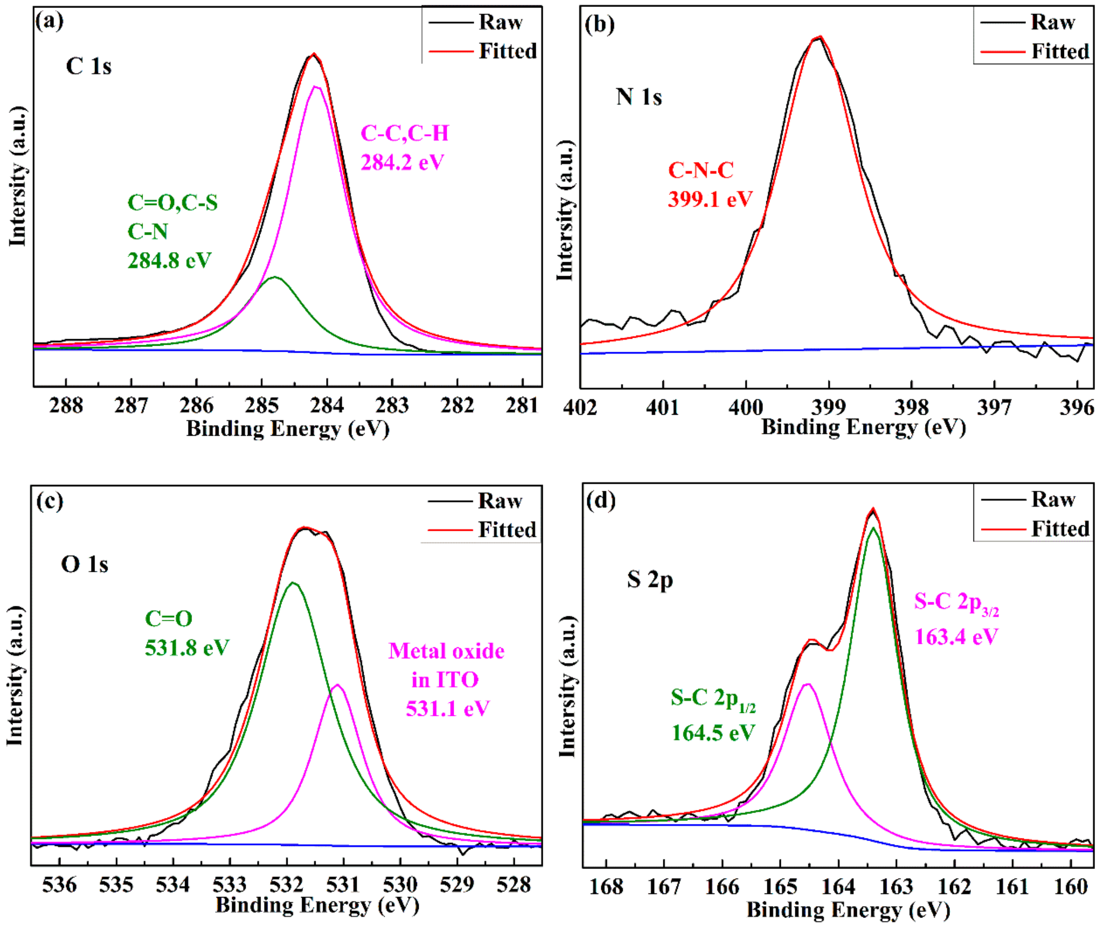

Figure S5. XPS spectra of polymer FLDPP: (**a**) C1s, (**b**) N1s, (**c**) O1s and (**d**) S2p.

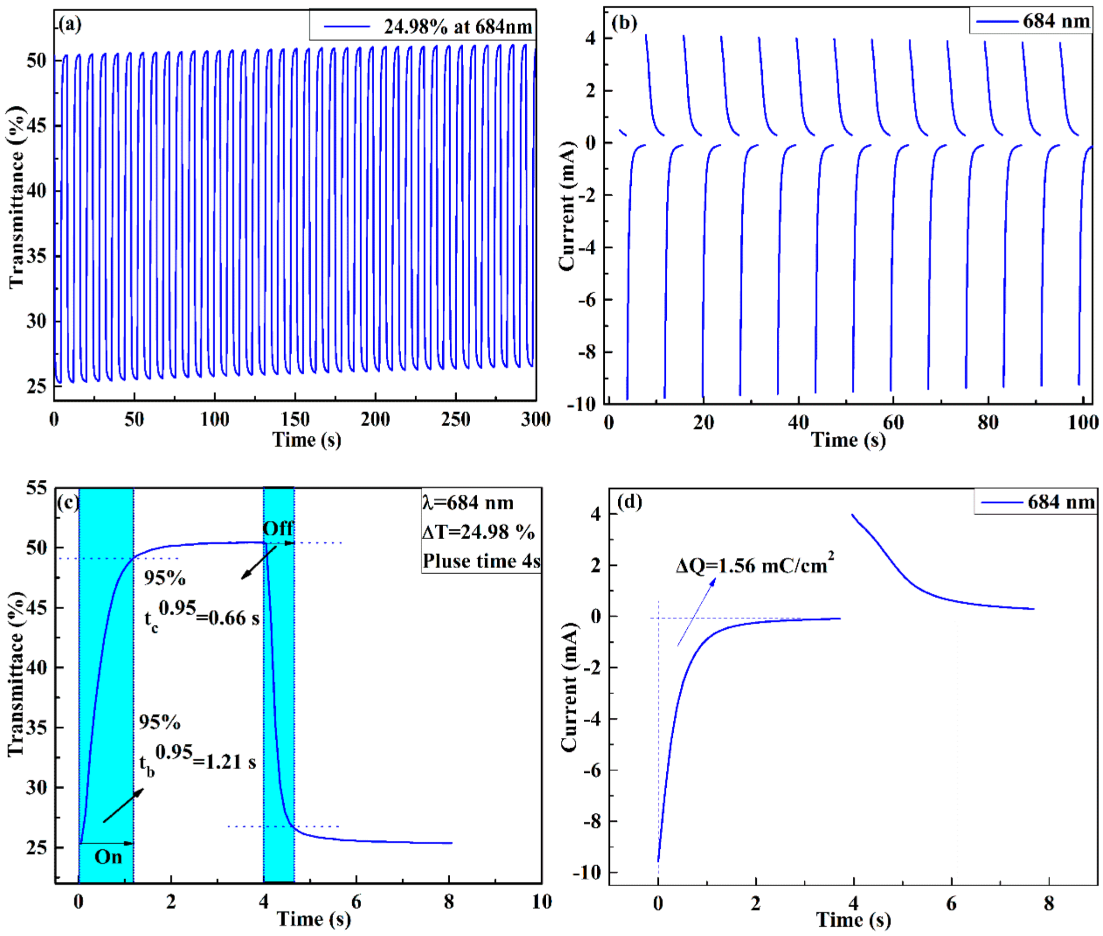

Figure S6. (**a**) Transmittance-time curve of BEDPP last for 300 s at 684 nm. (**b**)Current-time switching curve of BEDPP film between 0 V and 1.10 V in a time interval of 4 s. (**c**) The bleaching time (t_b) and the coloration time (t_c) of BEDPP at 684 nm. (**d**) The second cycle of current-time curve.

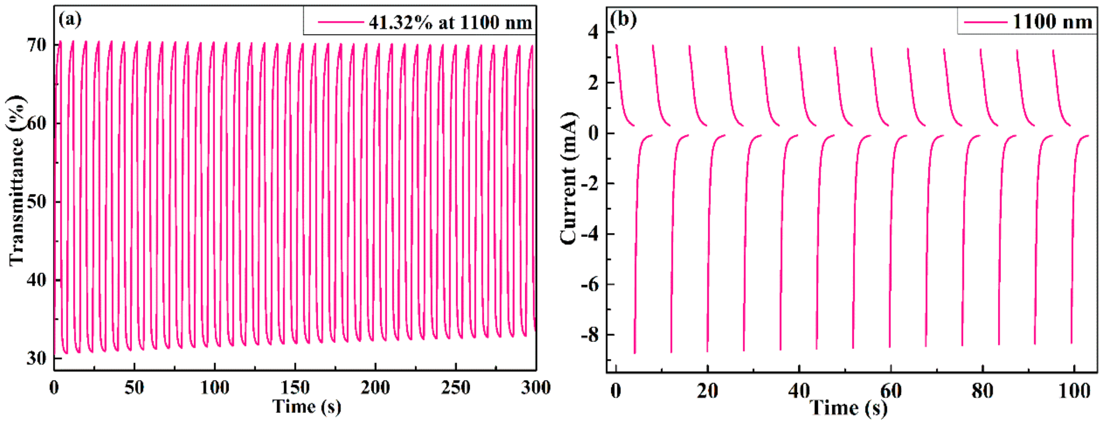

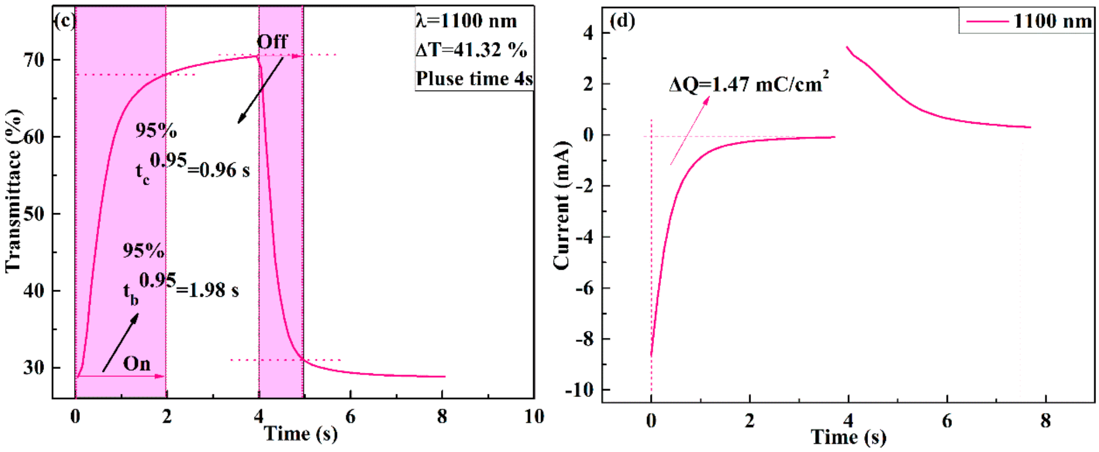

Figure S7. (**a**) Transmittance-time curve of BEDPP last for 300 s at 1100 nm. (**b**)Current-time switching curve of BEDPP film between 0 V and 1.10 V in a time interval of 4 s. (**c**) The bleaching time (t_b) and the coloration time (t_c) of BEDPP at 1100 nm. (**d**) The second cycle of current-time curve.

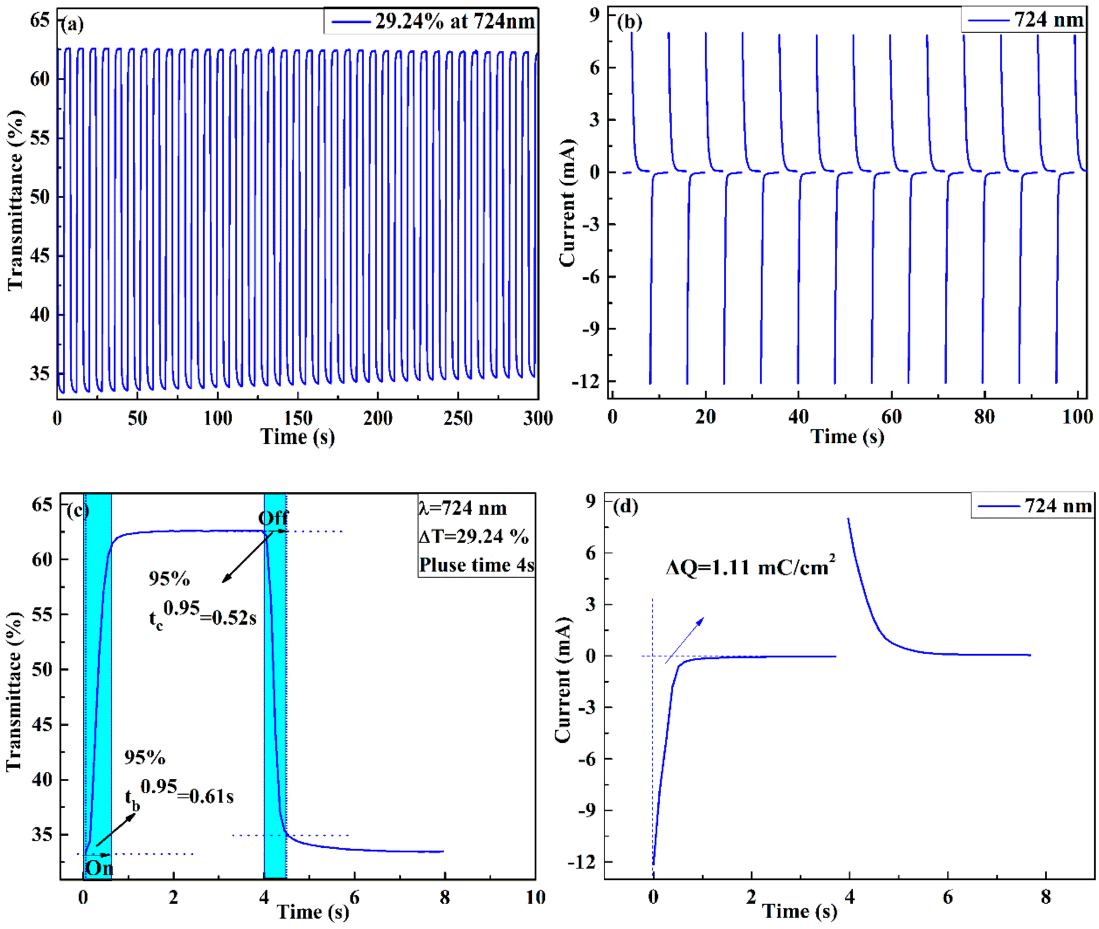

Figure S8. (**a**) Transmittance-time curve of FLDPP last for 300 s at 724 nm. (**b**) Current-time switching curve of FLDPP film between 0 V and 1.0 V in a time interval of 4 s. (**c**) The bleaching time (t_b) and the coloration time (t_c) of FLDPP at 724 nm. (**d**) The second cycle of current-time curve.

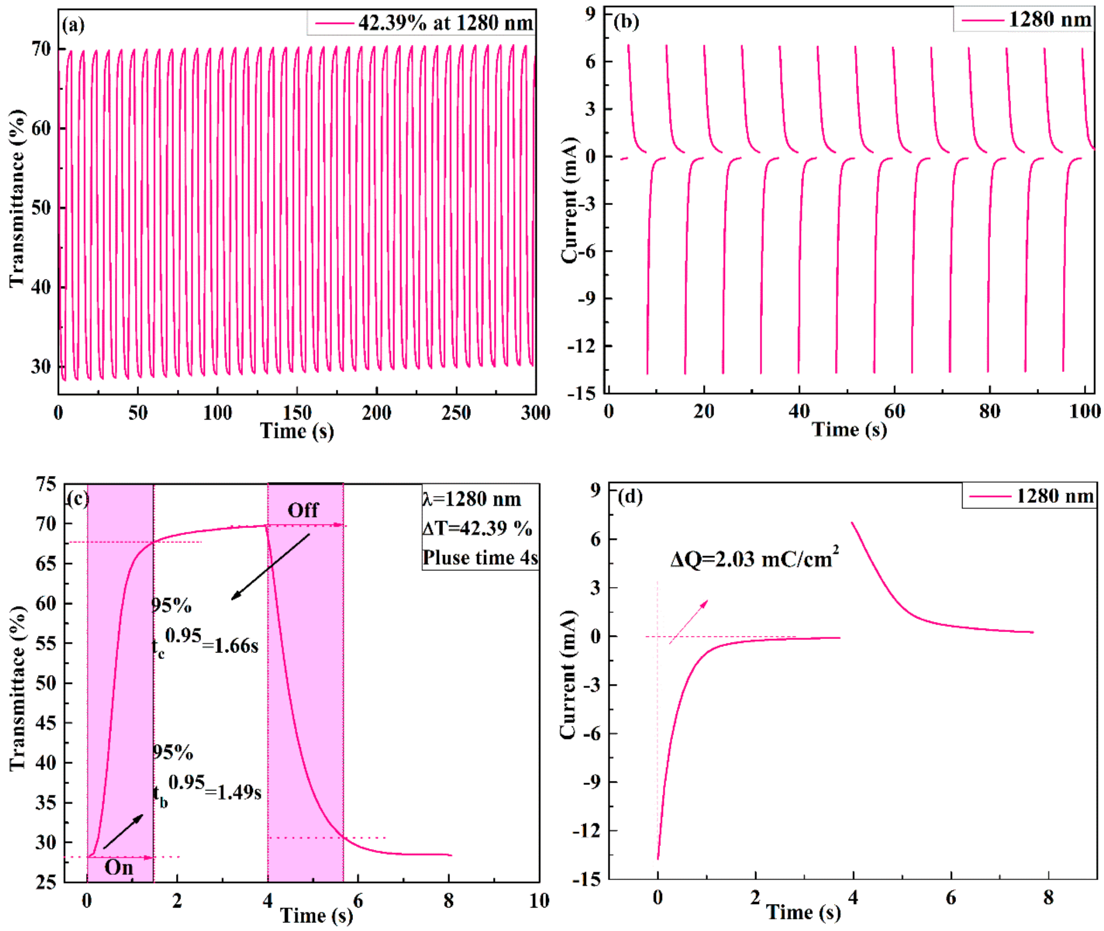

Figure S9. (**a**) Transmittance-time curve of FLDPP last for 300 s at 1280 nm. (**b**) Current-time switching curve of FLDPP film between 0 V and 1.0 V in a time interval of 4 s. (**c**) The bleaching time (t_b) and the coloration time (t_c) of FLDPP at 1280 nm. (**d**) The second cycle of current-time curve.

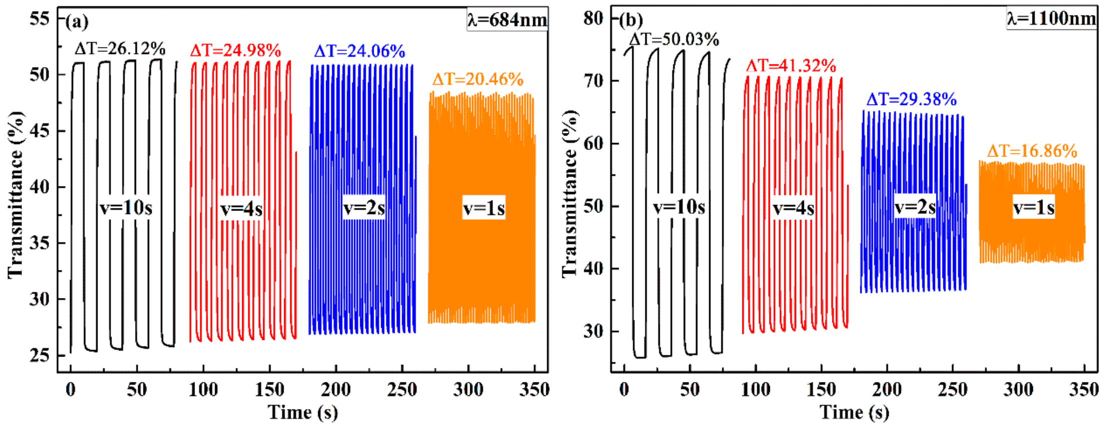

Figure S10. Electrochromic switching of BEDPP at 684 nm (**a**) and 1100 nm (**b**).

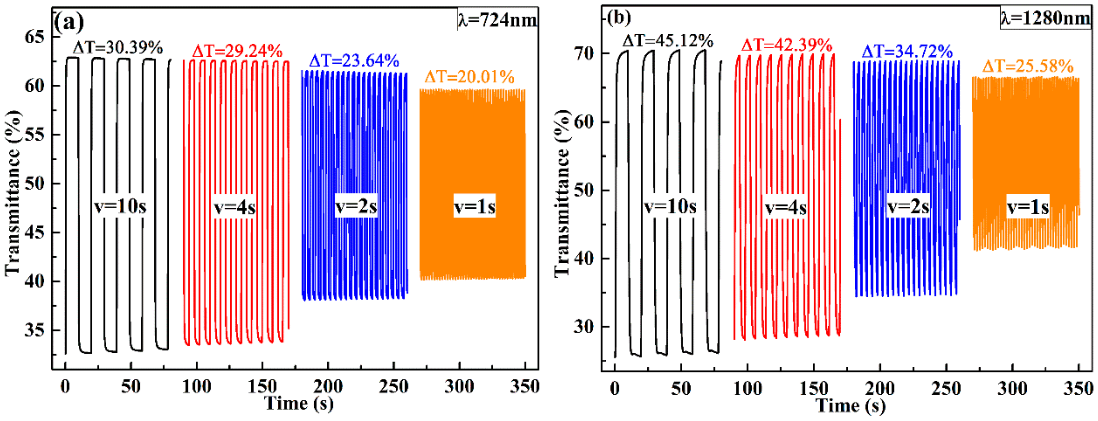

Figure S11. Electrochromic switching of FLDPP at 724 nm (**a**) and 1280 nm (**b**).

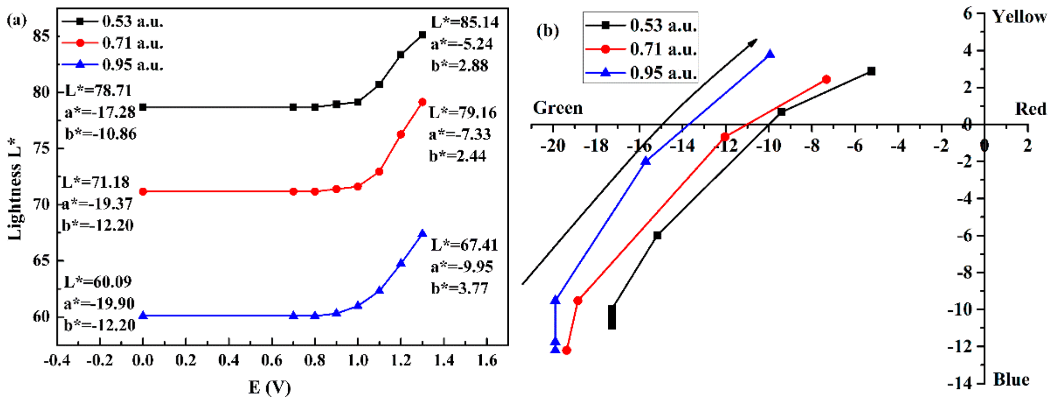

Figure S12. Lightness variation coordinates (**a**) and a* − b* values color space (**b**) of BEDPP during p-doping processing from neutral state to oxidized states based on three different thickness films. The direction of the arrows in (**b**) indicates the color changing trends of the polymer films.

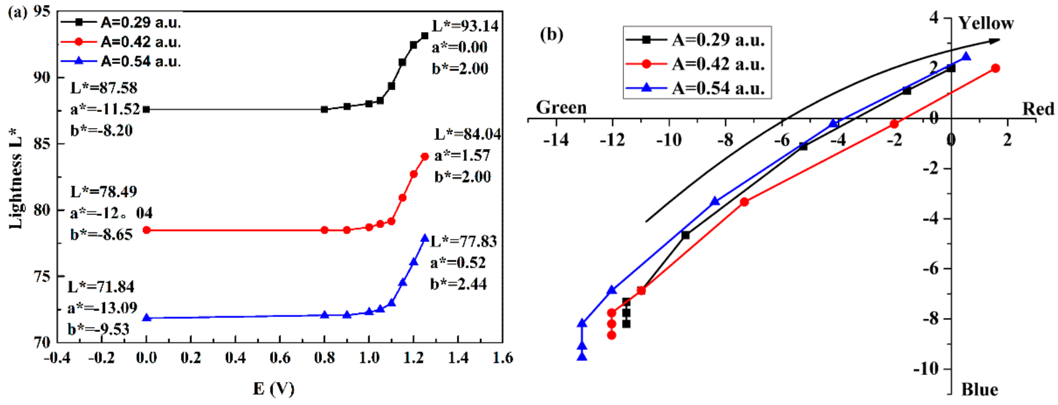

Figure S13. Lightness variation coordinates (**a**) and a* − b* values color space (**b**) of FLDPP during p-doping processing from neutral state to oxidized states based on three different thickness films. The direction of the arrows in (**b**) indicates the color changing trends of the polymer films.
